# Forest therapy as an intervention for depressive disorders: a review of the mechanisms and applications

**DOI:** 10.3389/fpubh.2026.1760446

**Published:** 2026-05-28

**Authors:** Fangbing Hu, Chengzhao Wu, Weiqing Liu

**Affiliations:** 1 Department of Landscape Architecture, Tongji University College of Architecture and Urban Planning, Shanghai, China; 2 Shanghai Pudong New Area Mental Health Center, Shanghai, China; 3 Tongji University School of Medicine, Shanghai, China

**Keywords:** depressive disorders, forest therapy, interdisciplinary study, intervention, mechanisms

## Abstract

Depressive disorders have gradually become a challenging public health issue worldwide, highlighting the need for accessible, non-pharmacological interventions. Forest therapy (or Shinrin-yoku) has emerged as a promising, low-cost, and sustainable approach. As a low-cost, low-risk, and sustainable environmental intervention, forest therapy harnesses nature’s bioactive and psychological effects to activate the body’s intrinsic healing mechanisms while averting medication costs and side effects. The field of forest therapy faces challenges, including unclear mechanisms, a lack of objective biomarkers, and fragmented clinical applications, with few stratified intervention protocols or standardized operating procedures. This review synthesizes evidence on the mechanisms and clinical applications of forest therapy for depressive disorders, summarizes biological mechanisms and social pathways, analyzes the pathways and mechanisms through which forest therapy, as an intervention, influences human health, and addresses current limitations, such as small sample sizes, and future directions, such as multicenter randomized controlled trials. Finally, this review proposed an integrated Mechanism Model of forest therapy as an adjunctive treatment for depressive disorders. Unlike previous studies, this review uniquely integrates forest environmental elements with core pathological targets to elucidate their interaction mechanisms. From a depressive disorders-centric perspective, this study calls for more clinical research and evidence-based design of therapeutic landscapes to support the integration of forest therapy into depressive disorder management.

## 1 Introduction

Depressive disorders, characterized by persistent low mood and loss of interest, are a leading cause of disability worldwide. The World Health Organization (WHO) recognizes depressive disorders as a serious public health issue. According to the latest data released by WHO in 2022, over 280 million people worldwide suffer from depressive disorders (1). In this context, forest therapy, also known as Shinrin-Yoku (forest bathing), has attracted substantial empirical and practical interest as a promising non-pharmacological approach for the search for complementary, non-pharmacological interventions.

Existing research on the environmental factors influencing this disease remains in its early stages, highlighting the critical role of environmental exposures as modifiable risk factors influencing depressive disorders risk and progression. There are emerging studies that have provided substantial epidemiological evidence linking diverse environmental factors, including air pollution, limited green space access, chronic noise exposure, adverse features of urbanization, unhealthy lifestyle behaviors, early life adversity, and internal toxicant burdens, to elevated risks of depressive symptoms and clinical depressive disorders over the years. A study has divided environmental exposures relevant to depressive disorders into three interrelated domains: general external exposures, specific external exposures, and internal exposures (2).

A recent study provides the evidence that depressive disorders are linked with environmental exposure, including air pollution, green space, noise exposure, climate change, urbanization and built environment, indoor environmental quality, lifestyle factors, psychosocial stress and early life adversity, heavy metals and endocrine disrupting chemicals, systemic inflammation and oxidative stress, and gut microbiota dysbiosis (3).

Among the above-mentioned environmental exposures, most studies focus on the negative impacts of pollutants (4) and on social environment impacts (5). Air pollution is among the most extensively investigated environmental exposures in relation to depressive disorders. Due to chemical emissions from various urban and industrial activities, air pollutants can have significant consequences for human neurodevelopment and the Central Nervous System (CNS) (6). Exposure to air pollutants during fetal life may be an important risk factor for neuro-developmental disorders and cause lifelong mental issues (7). Recent studies have also emerged to show that the biological impact of environmental factors in depressive disorders and other stress-related disorders is mediated by a variety of epigenetic modifications. These epigenetic modifications contribute to abnormal neuroendocrine responses, neuroplasticity impairment, neurotransmission dysfunction, and neuroglial dysfunction, all of which are involved in the pathophysiology of depressive disorders. This type of research indicates that environmental factors play an important role in mental health; however, most existing studies have focused on the negative effects of environmental exposure.

As for other types of environmental exposure, most are harmful to human health and can be categorized as environmental factors that degrade human health. In this study, we will discuss the environments that could benefit people with depressive disorders. Extensive evidence has confirmed that exposure to natural environments benefits physical and mental health, even aiding psychological well-being to some extent. One important aspect of nature is forests, or green space, in the urban context.

Forests and urban green spaces could keep people away from many of the harmful environments mentioned above, especially by significantly reducing air and noise pollution. Numerous studies have verified the functions of forests in reducing pollution and promoting human mental health. A case study from China showed that trees removed 1,261.4 tons of pollutants from the air in Beijing in 2002 (8). And a case from the United States also showed that the total amount of pollution removal in 2010 by trees and forests in the conterminous United States was 17.4 million t (range: 9.0 million t to 23.2 million t) with a human health value of $6.8 billion (range: $1.5 billion to $13.0 billion), which connecting the value of forests with human health (9). Based on the environmental studies, there is a substantial body of research on how forest and green spaces benefit to human health, which usually use the terms of “nature contact,” “forest healing,” “forest therapy,” “forest bathing,” “forest therapy,” “nature therapy,” and “nature exposure.”

In this study, we use the term forest therapy. Increasing studies have verified the positive effects of forest environments on both human physical and mental health. And theories such as Stress Reduction Theory and Attention Restoration Theory provide an initial theoretical basis for forest therapy. These posit that natural environments facilitate psychophysiological recovery from stress and mental fatigue. Emerging translational research has begun to delineate putative pathways, including modulation of the hypothalamic–pituitary–adrenal (HPA) axis, autonomic nervous system (ANS) balance, and inflammatory responses.

Forest therapy plays a complementary role in the treatment of certain diseases, and nature-assisted therapy (NAT) is a practical and suitable approach as a public health resource (10). A groundbreaking study in 1984 demonstrated that patients recovering from surgery in rooms overlooking a garden experienced faster recovery (11). Furthermore, forest therapy can enhance the human immune system. Several scientific studies have indicated a correlation between immune responses and exposure to nature, particularly in forests–an increase in the proportion of Natural Killing (NK) cells (12–14), anti-inflammatory, neuroprotective, and anti-tumor properties, purportedly linked to the inhalation of monoterpenes, and immune system stimulation associated with the inhalation of monoterpenes and an increase in both the number and activity of NK cells (15). From a broader perspective, forest therapy promotes interaction and social cohesion. In terms of social cohesion, the health benefits of exposure to nature depend on the recreational use of forests and green spaces (16). It fosters social connections, prosocial attitudes, the development of new relationships, community-building capabilities, empowerment, and reduces the risk of mental health issues. Conversely, social cohesion is associated with general health and mental well-being (17, 18). Sharing experiences with others may be a significant source of the benefits derived from forests (19). At the public health level, forest therapy can reduce morbidity and mortality. Regarding morbidity, a study found that proximity to green spaces reduced the population’s risk of dying from cancer, lung disease, or liver disease. Another study demonstrated that green spaces mitigate the effects of air pollution on blood pressure and diabetes (20).

More specifically, the psychological benefits of forest therapy encompass various aspects such as anxiety, depressive disorders, stress, mental restoration, emotional state, subjective well-being, and vitality, all of which are closely interconnected and interact with one another. The evaluation metrics primarily consist of various questionnaire scales (Table 1). Based on the past evidence, forest therapy mainly helps alleviate stress and restore attention. Simultaneously, it can reduce levels of anxiety and depressive disorders, improve sleep quality and cognitive function, and exhibit therapeutic effects on emotional and mental disorders. The short-term benefits of exposure to natural environments, such as forests and urban green spaces, for mental health have been well documented. These benefits include enhancing positive emotions, reducing subjective stress and negative emotions such as depressive disorders, fatigue, generalized anxiety, and uncertainty (21–25). Compared with urban environments, exposure to natural settings is more strongly associated with increased parasympathetic and reduced sympathetic nervous system activity (26, 27). Contact with natural spaces can also lower cortisol levels, promote attention restoration (28–30), and alleviate mental fatigue (31).

**Table 1:** Psychological benefits of forest therapy.

Psychological benefits	Psychological measurement tools	Health benefits
Emotional state	Profile of Mood States (POMS) Positive and Negative Affect Schedule (PANAS) Swedish Core Affect Scale (SCAS)	Contacting forests can enhance positive emotions, reduce negative emotions, and improve mood.
Depressive disorders	Montgomery-Åsberg Depressive Disorder Rating Scale (MADRS) Beck Depression Inventory (BDI) Self-Rating Depressive Disorders Scale (SDS)	Contacting forests can reduce depressive symptoms.
Anxiety	State–Trait Anxiety Inventory (STAI) Self-Rating Anxiety Scale (SAS)	Contacting forests can reduce anxiety levels.
Vitality	Subjective Vitality Scale (SVS)	Contacting forests can increase vitality.
Mental restoration	Restoration Outcomes Scale (ROS) Restorative Components Scale (RCS) Perceived Restorativeness Scale (PRS) Restorative Environment Scale (RES)	Contacting forests promotes mental restoration.
Attention	Stroop Color-Word Test Sustained Attention to Response Task (SART)	Contacting forests can improve attention.
Sleep quality	Pittsburgh Sleep Quality Index (PSQI)	Contacting forests can enhance sleep quality.
Stress	Perceived Stress Scale (PSS)	Contacting forests can reduce stress levels.
Fatigue	Self-Rating Fatigue Scale	Contacting forests can alleviate fatigue.
Subjective well-being	Semantic Differential Method (SD)	Contacting forests improves subjective perception.
Mental health	Warwick-Edinburgh Mental Well-being Scale (WEMWBS)	Contacting forests improves overall mental health status.

However, the translation of forest therapy into evidence-based practice for depressive disorders faces significant conceptual and methodological gaps currently. The therapeutic dose of forest exposure, for instance, encompassing duration, frequency, intensity, and environmental quality, remains poorly standardized and quantified. There are already studies that suggest the investigation of threshold effects, where benefits may only manifest after a certain level of exposure, a critical parameter for designing interventions (32–34). Moreover, the prevailing narrative overwhelmingly emphasizes benefits, potentially overlooking the dualistic nature of urban green spaces. Certain environmental factors, such as specific vegetation types that emit biogenic volatile organic compounds (BVOCs) that interact with urban pollutants, or high allergenic pollen loads, may inadvertently impose health burdens on susceptible individuals, establishing crucial boundary conditions for therapy.

Given the currently limited and heterogeneous evidence, which precludes a quantitative meta-analysis, this narrative review seeks to provide a novel conceptual synthesis by focusing on the human-nature interface. This study focuses on forest therapy as an intervention, systematically reviewing its pathways, mechanisms, and efficacy in alleviating depressive symptoms. Based on existing research, we aim to (1) elucidate the biological mechanisms, psychological mechanisms, and social pathways mechanisms, (2) analyze the pathways and mechanisms through which forest therapy as an Intervention influences human health, and (3) address research gaps such as current limitations and future directions. By synthesizing existing evidence, this study seeks to build a comprehensive mechanistic model of forest therapy as an intervention for depressive disorders and to provide a scientific foundation for incorporating forest therapy into management strategies for depressive disorders.

## 2 Apply the narrative review approach as a method

### 2.1 Rationale for a narrative synthesis

This review adopts a narrative review methodology. The decision was made not to employ a complete systematic review framework, for instance, following the PRISMA guidelines, or to conduct a meta-analysis, for two main reasons. Firstly, as mentioned in the introduction, the field of research investigating forest therapy specifically for depressive disorders is still developing. The author conducted an initial scoping of the literature and identified a limited number of studies that meet PRISMA criteria for a focused systematic review, which exhibit high heterogeneity in design, intervention, and measurement. In addition, the significant methodological studies varied in population, forest therapy protocols, control conditions, and outcome measures, making meaningful statistical pooling in a meta-analysis inappropriate and potentially uninformative at this stage.

As this review focuses on a highly interdisciplinary topic, a narrative approach allows for a more flexible and inclusive synthesis of the diverse evidence, enabling the authors to identify themes, patterns, and knowledge gaps without being constrained by the strict exclusion criteria of a systematic review, which might prematurely narrow the scope of learning from an emerging field.

### 2.2 Literature search and selection

Articles for this review were selected from SCOPUS, PubMed (MEDLINE), and Web of Science using a structured search, covering the inception through December 2025. The selection was based on title, year of publication, review of abstracts, and reading of the full text to assess relevance. The search strategy employed intentionally broad keywords: (“forest therapy” OR “Shinrin-Yoku” OR “forest bathing” OR “nature therapy”) AND (“depressive disorders” OR “mood disorder” OR “mental health”), to capture the interdisciplinary scope of the topic.

Additionally, reference lists of key articles and relevant reviews were hand-searched to identify further relevant studies. Study eligibility was determined primarily by relevance to the review’s aims rather than by rigid criteria; we prioritized empirical studies (of any design) in which forest exposure served as a primary intervention and reported outcomes related to depressive symptoms, mood, stress, or associated biomarkers. Literature on mechanisms, applications, or contextual factors was also included. Studies were excluded if they focused solely on general well-being without linking to depressive pathophysiology, or if the nature exposure was virtual or incidental to the study design.

Articles that did not directly address forest therapy or were not available in full text were excluded. This exclusion was applied to ensure methodological transparency and reproducibility, as the topic was a relatively new and interdisciplinary insight, and the authors could not find enough articles to conduct a systematic review. Most referenced papers are selected for their relevance to the association between forest therapy and improved mental health.

### 2.3 Data extraction and synthesis process

Consistent with the narrative review methodology, we did not perform a formal risk-of-bias assessment or data extraction for meta-analysis. Instead, the author first categorized all literature on the mechanical studies of depressive disorders, then systematically extracted key information from the included sources (study design, sample, intervention, key findings, proposed mechanisms). The author matched the extracted information one by one, which illustrates that forest therapy is related to its mechanism and how it works. We defined this process as bridging studies on depressive disorders to forest therapy, and the author team reviewed and discussed the relationship between environmental effects and human body responses to identify convergent themes, conflicting evidence, and research gaps. The synthesis is presented thematically, guided by the hypothesis of depressive disorders identified in the literature.

## 3 Pathophysiological targets in depressive disorders and the multimodal mechanisms of forest therapy

Understanding the mechanisms through which forest therapy may benefit depressive disorders requires first delineating the key pathophysiological processes involved in depressive disorders, which in turn serve as potential intervention targets. Regarding the pathogenesis of depressive disorders, current research primarily focuses on mechanisms such as the monoamine hypothesis (35), alterations in the hypothalamic–pituitary–adrenal (HPA) axis (36), inflammation (37), neuroplasticity, changes in brain function and structure (38), as well as environmental stressors (39), and gene–environment interactions (5). These studies highlight the significant role of environmental factors in the development of depressive disorders. The onset of depressive disorders is closely linked to dysregulation of the neuroendocrine-immune network, and disturbances in this network can amplify or sustain depressive symptoms through multiple mechanisms. Forest therapy is hypothesized to exert its effects by modulating these very systems.

As a nature-based intervention, forest therapy has demonstrated significant potential in recent years as an adjunctive treatment for depressive disorders (40, 41). Existing research indicates that the mechanisms through which forest therapy aids in alleviating depressive symptoms operate across physiological, psychological, and social dimensions (42). These include enhancing neuroplasticity, regulating neuroimmune responses, reducing inflammation, and improving antioxidant capacity, among others. Collectively, these mechanisms contribute to its clinical relevance in terms of comprehensive therapeutic efficacy.

Table 2 summarizes the biological pathways through which forest therapy exerts its therapeutic effects. Forest therapy induces multi-system responses upon environmental enrichment, physical activity, cognitive stimulation, and mindfulness practice. On a neural level, it elevates brain-derived neurotrophic factor (BDNF) and promotes hippocampal expansion (43, 44); in endocrine regulation, it suppresses hypothalamic–pituitary–adrenal (HPA) axis activity, effectively mitigating chronic stress through mindfulness, physical activities and some natural products in the forest (45, 46); immunologically, the Phytochemicals in the forests could downregulate pro-inflammatory cytokines and inhibits NLRP3 inflammasome activation, alleviating neuroinflammation. Furthermore, antioxidant defense, such as by boosting superoxide dismutase (SOD) activity, and rebalancing autonomic nervous function through increased parasympathetic tone, could be reinforced by the Phytochemicals, thereby supporting neural integrity (47). Notably, the microbiota–gut–brain axis in the human body also plays a significant role in human health. The microbial metabolites, such as short-chain fatty acids, modulate tryptophan metabolism, revealing a novel pathway through which natural environments can remotely influence neurotransmitter regulation (48).

**Table 2:** Biological pathways and therapeutic effects of forest therapy.

Biological pathways	Therapeutic effects
Neuroplasticity increase (117)	Improve cognitive function and prevent neurodegeneration.
HPA axis regulation	Alleviation of chronic stress responses
Anti-inflammatory effect	Mitigation of neuroinflammation
Antioxidant protection	Reduction of oxidative stress damage
Autonomic balance	Promotion of physical and mental relaxation
Microbiota-gut-brain axis regulation	Modulation of tryptophan metabolism and neurotransmitter balance

Regarding depressive symptom improvement, a Korean study found that participants in the forest therapy group showed greater physiological improvement than those in the control group, as indicated by significant increases in some HRV measures. Forest therapy also contributed to a significantly greater decrease in work-related stress symptoms and a significantly greater improvement in health-related quality of life and mood states compared to participants in the control group (49). Regarding physiological biomarkers, a study on childhood and adolescent cancer survivors observed a decrease in cortisol of 11% in cases and 37% in controls (cases: *p* = 0.260; controls: *p* = 0.028), a decrease in *α*-amylase of 75.93% in cases and 51.73% in controls (cases: *p* = 0.008; controls: *p* = 0.612). The differences were statistically significant (*p* < 0.05) for cortisol levels in controls and α-amylase levels in cases (50). The observed differences corresponded to decreases in cortisol and α-amylase levels during exposure to forest environments, consistent with the findings of Hunter et al. (51). At the molecular level, a 2025 JAMA Psychiatry study reported significant reductions in pro-inflammatory cytokines IL-6 and TNF-*α*, along with elevated BDNF levels—a neuroplasticity marker—observed in both human and mouse experiments (52). Collectively, these findings demonstrate that forest therapy simultaneously ameliorates depressive disorders across three dimensions: psychological symptoms, physiological stress responses, and molecular pathology.

### 3.1 Neurological dysregulation and potential modulation by forest environments

Current research indicates that dysregulation of the nervous system is the root cause of emotional and cognitive symptoms, primarily manifested in the following aspects.

Depressive disorders are characterized by two core pathophysiological mechanisms: monoamine neurotransmitter deficits and neuroplasticity impairment. The depletion of monoamines (5-HT, NE, DA) leads to abnormal signal transduction in the prefrontal cortex and limbic system, manifesting as core depressive symptoms like low mood and anhedonia. This process is exacerbated by immune-inflammatory activity, in which pro-inflammatory cytokines like IL-6 activate IDO (indoleamine 2,3-dioxygenase), accelerating tryptophan degradation and reducing serotonin synthesis (53). Concurrently, neuroplasticity dysfunction occurs with BDNF deficiency, triggered by chronic stress-induced cortisol elevation that causes hippocampal atrophy and cognitive impairments. The immune system further aggravates this via TNF-α-mediated suppression of BDNF (brain-derived neurotrophic factor) expression (54). Forest therapy may counteract these pathways through multiple mechanisms: phytoncides could normalize monoamine levels by reducing IDO activation, which is related to depressive disorder (55). In addition, negative air ions play a physiological regulatory role, directly stimulating the nervous reflex and the humoral system (56, 57). Further, the stress-reducing forest environment lowers cortisol levels, thereby protecting hippocampal neurons (58). All these factors collectively illustrate how nature exposure can modulate the neuro-immune axis in depressive disorders [Key abbreviations: 5-HT = serotonin; NE = norepinephrine; DA = dopamine].

Forest therapy has been demonstrated to effectively reduce the incidence of neurological abnormalities, with one of its core mechanisms being the promotion of neurogenesis to maintain brain health (59, 60). Neurogenesis refers to the continuous process by which neural stem cells generate new neurons in specific brain regions. One of them is the hippocampal dentate gyrus, a process that persists throughout the human lifespan (61). Research indicates that patients with depressive disorders show not only reduced neuron counts in the hippocampal dentate gyrus but also significant impairment in neuronal differentiation processes. The forest environment enhances brain-derived neurotrophic factor (BDNF) levels, promoting neuroplasticity across multiple dimensions to improve cognitive function and reduce the risk of neuropsychiatric disorders (62–64). A study found that walking for 12 weeks increased BDNF levels, which support brain cell growth and cognitive function (65). Shin et al. found that 12-week forest-slope movement and phytoncides affected brain-derived neurotrophic factor, which accelerates the development of brain cells and supports cognitive function (66).

The regulation of BDNF through forest therapy operates via four potential pathways. Firstly, mild aerobic exercise during forest activities (such as walking) induces calcium influx, activating the CaMKII and CREB signaling pathways to upregulate BDNF gene expression (67, 68). The lactate produced during exercise can cross the blood–brain barrier to directly stimulate BDNF synthesis in the hippocampus (69). Secondly, forest exposure reduces chronic stress by activating the parasympathetic nervous system, thereby decreasing cortisol secretion and, in turn, alleviating its inhibitory effect on BDNF production (70). Neuroimaging studies reveal that forest walks significantly reduce amygdala activity associated with stress responses (71). Thirdly, phytoncides, the volatile organic compounds released by trees in the forest, exert direct neuroprotective effects by reducing inflammation and enhancing antioxidant capacity, thereby maintaining BDNF stability (72, 73). Clinical observations demonstrate synchronized increases in both BDNF levels and natural killer (NK) cell activity following inhalation of forest air (74, 75). Lastly, beneficial microorganisms in forest soil may influence BDNF through the gut-brain axis, where short-chain fatty acids (SCFAs) stimulate the Vagus nerve to upregulate hippocampal BDNF while reducing systemic inflammation that would otherwise suppress BDNF (76, 77). This multi-targeted approach positions forest therapy as a comprehensive, non-pharmacological intervention for maintaining neurological health.

### 3.2 Neuroendocrine dysfunction and induced stress reduction by forest therapy

Another central hypothesis in the pathogenesis of depressive disorders involves endocrine dysregulation of the hypothalamic–pituitary–adrenal (HPA) axis, primarily manifested as sustained elevations in cortisol levels and circadian rhythm disruption (78, 79). Due to reduced sensitivity of hippocampal glucocorticoid receptors (GR), their inhibitory effect on the HPA axis is impaired, leading to chronically elevated cortisol levels. This failure in negative feedback regulation suppresses neurogenesis (which may manifest as hippocampal volume reduction observable on MRI). It enhances amygdala activity (resulting in amplified anxiety and negative emotions), explaining why most cases of depressive disorders worsen without intervention. Furthermore, disrupted cortisol rhythms lead to abnormal melatonin secretion, contributing to the sleep disturbances (early awakening/insomnia) (80).

The therapeutic action of forest therapy could be the normalization of stress physiology in human bodies. The capacity of forest environments to promote physiological relaxation and dampen HPA axis overactivity is one of the most consistently documented effects. Studies across various protocols show significant reductions in salivary and serum cortisol following forest therapy sessions compared with urban control settings (81). This is supported by neuroimaging research showing that exposure to natural scenes reduces amygdala activity, a key driver of the stress response (71, 82). The combination of sensory inputs, fractal visual patterns, natural sounds masking urban noise, and calming scents appears to signal safety to the brain, reducing limbic activation and its downstream effects on the sympathetic nervous system and the HPA axis. This shift is further evidenced by increased heart rate variability (HRV), indicating enhanced parasympathetic (rest-and-digest) tone and a rebalancing of the autonomic nervous system toward a state incompatible with chronic stress (26, 42).

### 3.3 Immune-inflammatory activation and the anti-inflammatory potential of forest therapy

Another proposed mechanism of depressive disorders is the inflammation hypothesis, which posits that inflammatory markers are closely linked to the onset, progression, and treatment response of depressive disorders. In 2024, an analysis of over 1 million individuals from Sweden’s AMORIS cohort and the UK Biobank revealed a significant association between blood inflammatory markers and the risk of mental disorders. The study found that individuals with levels of white blood cells (WBCs), haptoglobin, and C-reactive protein (CRP) above the median had increased risks of 11, 13, and 2%, respectively, of developing any mental disorder. Conversely, those with immunoglobulin G (IgG) levels below the median exhibited an 8% reduced risk. These findings provide novel biomarkers for early detection of mental disorders and open new perspectives on their pathogenesis. The study further employed Mendelian randomization analysis to examine the relationship between inflammatory markers (WBCs, haptoglobin, IgG, CRP, platelets, albumin, and WBC subtypes) and depressive disorders, revealing a positive genetic correlation between CRP and depressive disorders (83). Notably, a recent study provided additional confirmation, demonstrating significantly elevated levels of multiple inflammatory biomarkers in the peripheral blood of depressive disorders, including pro-inflammatory cytokines interleukin-6 (IL-6) and tumor necrosis factor (TNF), as well as the clinically common acute-phase reactant CRP. Moreover, a link has been observed between autoimmunity and depressive disorders, with patients frequently exhibiting overactive Th1 (pro-inflammatory) responses and a high comorbidity rate with autoimmune diseases like Hashimoto’s thyroiditis. Collectively, these findings highlight the critical role of inflammatory pathways in the pathological mechanisms of depressive disorders, offering new biological insights for diagnosis and treatment (52).

However, the underlying mechanisms connecting inflammatory biomarkers to depressive disorders remain unclear. Potential explanations include blood–brain barrier disruption, microglial activation, neurotransmission impairment, hyperactivity of the hypothalamic–pituitary–adrenal (HPA) axis, alterations in gut microbiota, and other neuro-immune interactions. Further research is needed to elucidate these mechanisms (118, 119).

One definition of neuroinflammation from the literature defines it as an immune response characterized by the abnormal activation of glial cells and the release of inflammatory factors when the body is exposed to external stimuli (84). Current research indicates that modern urban living conditions, including exposure to air pollution and unhealthy lifestyle habits that lead to suboptimal health, are associated with neuroinflammation. Environmental factors may trigger inflammatory responses, and chronic inflammation can exacerbate tissue damage and disease progression. Notably, depressive disorders involve extensive neuroinflammatory responses across multiple brain regions, including the hippocampus, olfactory bulb, and prefrontal cortex.

Research findings demonstrate that microglial activation and altered levels of various inflammatory factors, for instance, interleukin-1*α* (IL-1α), interleukin-1β (IL-1β), interleukin-6 (IL-6), and tumor necrosis factor-α (TNF-α) (85–89) constitute one of the primary mechanisms underlying depressive disorders’ pathogenesis, which is related to the forest environment. Studies revealed that forest-derived components can effectively reduce inflammatory responses in the human body. Firstly, sensory stimulation from the forest activates the human parasympathetic nervous system, lowering cortisol levels and effectively reducing systemic inflammation (42). Secondly, the negative oxygen ions in forest air decrease free radicals, mitigating oxidative stress (a key inflammatory trigger), while regulating the autonomic nervous system to suppress sympathetic overactivity (chronic stress exacerbates inflammation) (90). Thirdly, trees (such as pine, cedar, and cypress) release volatile organic compounds, including α-pine and limonene, with demonstrated antibacterial and antifungal properties. Studies show that inhalation of these compounds reduces pro-inflammatory cytokines (IL-6, TNF-α) while enhancing NK cell activity, thereby alleviating chronic inflammation (91, 92). In addition, people often engage in light physical activities in forests, such as walking and yoga, which could improve blood circulation, increase the secretion of anti-inflammatory cytokines, such as IL-10, and reduce visceral fat, a known source of inflammatory factors (93–95). Last but not least, contact with forest soil and plants may increase exposure to beneficial microbes that modulate immune balance through the “immune-microbiome axis,” thereby preventing excessive inflammatory responses, which could, in turn, affect depressive disorders (96, 97).

**Figure 1:**
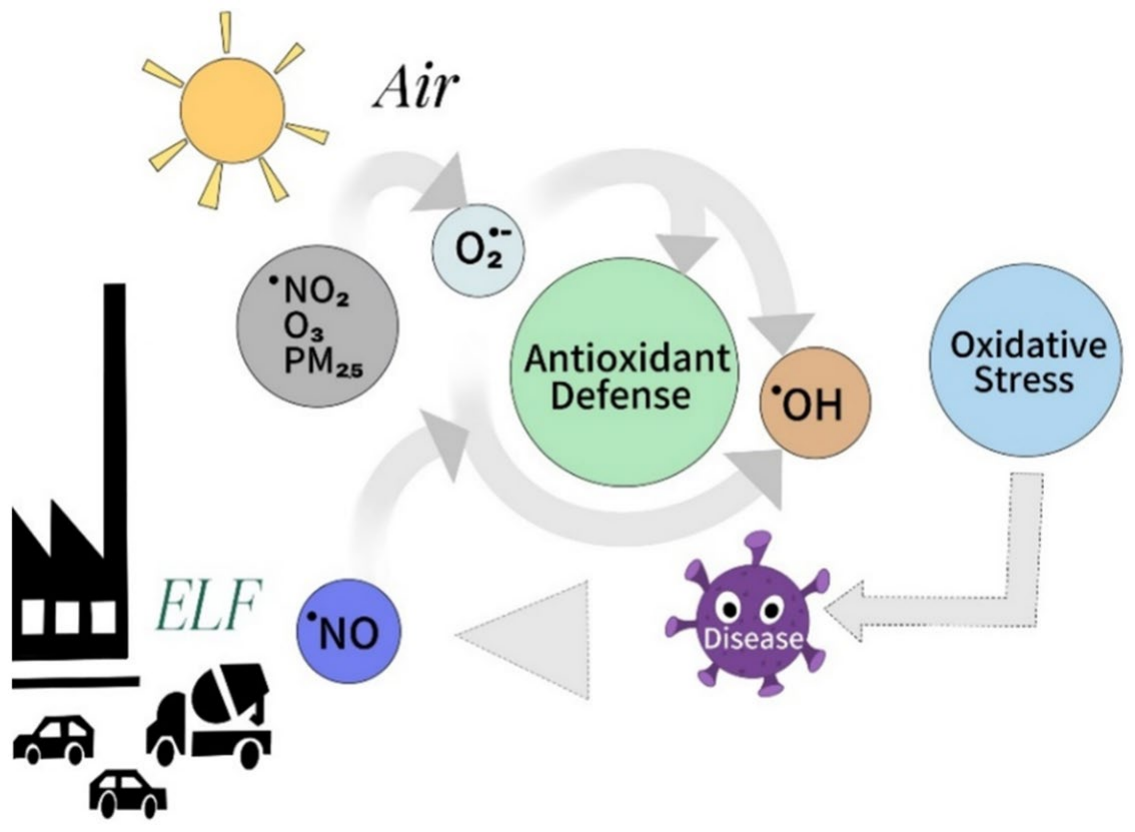
The oxidative stress caused by the environment (ELF, Extremely low frequency electromagnetic fields; self-drawn by the author).

Another indirect factor related to inflammation would be oxidative stress. Oxidative stress refers to an imbalance between oxidation and antioxidation in the body (98), often accompanied by inflammation and further damaging neurons, exacerbating this pathological process. Due to its high oxygen consumption, elevated lipid content, and relatively weak antioxidant capacity, the brain is particularly vulnerable to oxidative damage. Oxidative stress is a key contributor to neurodegeneration and has been closely linked to depressive disorders (Figure 1).

Forest therapy could mitigate oxidative stress primarily through negative air ions present in forests. Research indicates that negative ions alleviate oxidative stress via three main mechanisms. Firstly, direct neutralization of free radicals. Studies have found that negative ions can increase superoxide dismutase (SOD) activity (99). Moreover, negative air ions could indirectly activate the antioxidant pathways (100). Negative ions stimulate the nuclear factor E2-related factor-2 (Nrf2) pathway, activating the Keap1-Nrf2-ARE signaling axis, which upregulates antioxidant enzymes such as SOD and glutathione peroxidase (GPx) (101, 102). The Nrf2 is a crucial transcription factor in cellular antioxidant responses. Research using Nrf2−/− mice demonstrated that Nrf2 deficiency exacerbates PM2.5-induced depressive-like behaviors by disrupting neurotransmitter balance (103). Compared to wild-type mice, Nrf2 knockout significantly increased pro-inflammatory cytokine expression and NLRP3 inflammasome activity, suggesting that Nrf2-mediated oxidative stress regulation may influence depressive disorders via the NLRP3 inflammasome pathway (103). Negative ions also suppress NF-κB, reducing NADPH oxidase (NOX) activation and limiting free radical production at the source (104). Lastly, negative ions enhance mitochondrial electron transport chain efficiency, thereby minimizing electron leakage, a significant source of free radicals, particularly in high-energy-demand tissues such as the brain and heart (105). In a nutshell, forest therapy may address this inflammatory component through both indirect and direct pathways.

### 3.4 Multisystem synergy as a therapeutic rationale for forest therapy

Critically, the systems described above function correctly in isolation. They interact to form a vicious, self-reinforcing cycle that sustains depressive pathology. Chronic stress activates the HPA axis and sympathetic nervous system, elevating cortisol and promoting inflammation. Inflammatory cytokines, in turn, further dysregulate the HPA axis, inhibit BDNF production, and contribute to neurotoxicity. Moreover, reduced BDNF and neuronal damage impair cognitive function and stress resilience, perpetuating a sense of overwhelm and keeping the stress response activated. This interconnectedness explains the chronicity of depressive disorders and underscores the limitation of interventions targeting a single pathway.

The proposed therapeutic power of forest therapy may lie precisely in its capacity for gentle, simultaneous multi-system modulation, making it uniquely suited to disrupt this vicious cycle. Unlike a pharmacological agent with a single primary target, forest therapy delivers a package of therapeutic stimuli, including sensory engagement like sight, sound, and smell, mild physical activity, psychological restoration, and positive social context. This package induces parallel responses; for instance, it downregulates the hyperactive stress response (HPA axis, sympathetic tone), promotes anti-inflammatory and antioxidant activity, creates conditions conducive to neuroplasticity (via stress reduction and potential BDNF support), and facilitates psychological restoration through attention restoration and stress reduction theories, which themselves positively influence the biological states.

This multisystem synergy is the central thesis for forest therapy’s mechanism in depressive disorders. By concurrently nudging several dysregulated systems toward balance, it may weaken the links in the pathological cycle at multiple points, promoting a shift toward homeostasis. Future research with rigorous designs that include biomarkers from these different systems will be crucial to validating this integrative model and moving forest therapy from a promising practice to an evidence-based component of integrative mental health care.

## 4 Social and contextual pathways in forest therapy for depressive disorder

Even though the biological and psychological pathways described in section 3 are crucial, they do not operate in a vacuum. Depressive disorders are profoundly influenced by, and in turn influence, a person’s social behavior and environmental context. Social withdrawal, loneliness, diminished self-worth, and experienced stigma are not mere consequences but core features of the disorder that interact bidirectionally with biological dysregulation (106).

As an activity that often involves groups, forest therapy inherently engages social and contextual pathways that may directly address these dimensions and amplify the benefits of biological modulation. This study frames them as active therapeutic components rather than incidental benefits. The social mechanisms of forest therapy as an adjunctive intervention for depressive disorders are primarily manifested through multiple pathways. This study summarizes the literature on the social mechanisms provided by forest therapy as an adjunctive treatment for depressive disorders.

### 4.1 Forest therapy helps with facilitating social reconnection in a low-threat environment

During forest therapy sessions, natural interactions foster social bonds among individuals. A core behavioral manifestation of depressive disorder is social withdrawal, the avoidance of social interactions due to anticipatory anxiety, anhedonia, or feelings of worthlessness (106). The forest environment acts as a normative, non-clinical, and de-stigmatizing setting for social interaction. Unlike a therapy room, a forest walk is a socially valid activity that reduces the perceived “patient” role and associated evaluation anxiety. The shared focus on the external environment provides a structured, low-demand framework for parallel and joint engagement. This can facilitate initial social reconnection and reduce psychological burden, helping break the cycle of isolation. Qualitative studies note that the shared nature experience becomes a neutral, positive topic of conversation, easing social interaction among participants (107). Moreover, forest therapy often requires participants to engage in pro-nature activities to enhance their sense of responsibility and executive function, aiding the restoration of social capabilities (108). Some programs incorporate vocational training components to improve further occupational skills and social adaptability, which are essential for them to rebuild social connections.

### 4.2 Forest therapy fosters community support and enhances self-efficacy

Depressive disorders often erode an individual’s sense of competence and agency. The loss of social roles and supportive networks further diminishes self-efficacy, a key predictor of recovery. Forest therapy programs often incorporate simple, goal-oriented group activities such as collaborative mapping, gentle gardening, or a shared mindfulness walk. Completing these tasks within a supportive group provides mastery experiences that can counteract feelings of helplessness. The group format inherently builds a temporary therapeutic community, offering peer support, normalization of experiences, and a sense of belonging, which is a powerful antidote to the alienation of depressive disorders (109). This “collective efficacy” experienced in a group achieving a shared, positive goal in nature can reinforce individual self-efficacy.

### 4.3 Forest therapy restores meaning and identity in the cultural and ecological connection

Depressive disorders can strip life of meaning and pleasure. Reconnecting with something larger than oneself, be it nature, local ecology, or cultural heritage. Can be profoundly therapeutic. Forest therapy can facilitate meaning-making through ecological connection. Learning about local flora and fauna, understanding seasonal cycles, or participating in light stewardship activities, for instance, planting native species, fosters a sense of relatedness to the living world. This can counter the inward focus and nihilism of depressive disorders. Furthermore, in culturally appropriate programs, integrating local indigenous or traditional forest knowledge can strengthen cultural identity and continuity, offering another layer of meaning and resilience (110). This process of “re-embedding” oneself within a meaningful ecological and cultural narrative can challenge the constricted self-view common in depressive disorders.

Forest therapy constructs a “social reintegration” healing field by combining natural social catalysts, culturally empowered identity formation, and sustained community support. Its core social mechanism lies in re-embedding isolated individuals into a supportive network interwoven with nature and culture, thereby alleviating depressive symptoms and facilitating functional recovery. Future integration with technologies such as VR and cultural innovation could position this model as a pivotal direction for societal mental health interventions.

### 4.4 Forest therapy could be more acceptable by addressing stigma and promoting equitable access

Stigma in relation to mental disorders has been identified and recognized as a major concern across societies worldwide (111). Furthermore, the benefits of nature are not equitably distributed. Access to high-quality, safe green space is influenced by socioeconomic status, geography, and disability.

However, the social benefits theorized here presuppose equitable access. If therapeutic forest programs are only available in affluent areas or pristine, remote forests, they risk exacerbating health inequities. Therefore, the social mechanism is contingent on equitable implementation. A study from Hangzhou, China, has revealed that it is crucial to investigate environmental equity among groups at a small scale, where the prevalence of intersections and similar daily exposure patterns across populations highlights broader equity concerns across urban landscapes. At this scale, various vegetation configurations and building forms would influence PM2.5 dispersion and thereby lead to disparities in population exposure. This study highlighted that forest therapy, as an intervention that may be applied in urban design, should consider the complex interactions among vegetation patterns, pollution dispersion, and population exposure (112).

This necessitates the intentional design and distribution of therapeutic landscapes in accessible urban and peri-urban locations, taking into account public transport, safety, and universal design principles. Research and practice must actively address these contextual moderators to ensure the social pathway is inclusive.

### 4.5 Synthesizing the social-contextual pathways as integral to the therapeutic mechanism for forest therapy

The social and contextual dimensions of forest therapy are not secondary “add-ons” but integral, active ingredients that interact synergistically with biological and psychological pathways. The supportive social context can enhance stress reduction, bolstering the biological effects mentioned above. The experience of mastery and connection can improve mood and motivation, creating a positive feedback loop. Conversely, the physiological calm induced by the forest environment likely lowers social anxiety, making social interactions more feasible and positive.

In conclusion, forest therapy constructs a unique “bio-psycho-social-ecological” niche for intervention. It leverages the inherent social catalysts of group activity in a calming environment, combines them with meaningful engagement with the natural world, and uses this combination to directly counter the social isolation, diminished identity, and stigmatization that are central to the experience of depressive disorders. Future program design and research should explicitly measure these social-contextual outcomes and their interactions with biological measures to capture the intervention’s impact fully.

## 5 Critical synthesis, limitations, and future research

This review has advanced the thesis that forest therapy represents a promising multi-modal, system-level intervention for depressive disorders. By integrating evidence across biological, psychological, and social domains, we propose that its potential efficacy stems from its capacity to simultaneously and gently modulate several interconnected dysregulated pathways that characterize depressive disorders. This final chapter synthesizes these integrated findings, critically appraises the strength of the current evidence and the limitations of this synthesis, and charts a translational roadmap to advance the field from promising concept to evidence-based practice.

### 5.1 Synthesis of an integrated mechanism model

The pathophysiology of depressive disorders is best understood as a vicious cycle of dysregulation across neuroendocrine (HPA axis hyperactivity), immune-inflammatory (elevated cytokines), neural (reduced plasticity), and autonomic (sympathetic dominance) systems, compounded by psychological distress and social withdrawal. Forest therapy, through its multi-sensory engagement with natural environments, appears to interact with this cycle at multiple nodes. Biologically, it provides consistent evidence of reduced cortisol and sympathetic tone, with plausible pathways to mitigate inflammation and create a milieu supportive of neuroplasticity. Psychologically, it facilitates attention restoration and stress reduction, which likely initiate and reinforce positive biological changes. And socially, it provides a low-threat context for social reconnection and meaning-making, directly countering core psychosocial deficits in depressive disorders.

The proposed synergy is key, as no single pathway is likely responsible for the clinical effects. Instead, the concurrent modulation across systems may disrupt the self-perpetuating pathological cycle, promoting a shift toward homeostasis. This integrative model positions forest therapy not as a simple “nature pill,” but as a complex intervention whose active ingredients include specific environmental features, induced activities, and the social context of delivery.

### 5.2 Limitations of the evidence and this review

While the proposed model is theoretically coherent, its empirical foundation requires strengthening amid significant limitations. Firstly, there are many limitations in the primary research literature in this field, and most studies exhibit methodological heterogeneity and weak designs. Many studies are small-scale (N < 50), employ wait-list or passive controls, and lack long-term follow-up (113). Few use active comparators to isolate the unique “forest effect” from confounding factors such as exercise, being outdoors, or group interaction (27, 114). Additionally, the mechanistic evidence in existing research is often indirect, with claims about mechanisms such as increased BDNF or reduced specific cytokines frequently extrapolated from animal studies, *in vitro* work, or studies in healthy populations (115). Direct, simultaneous measurement of a panel of relevant biomarkers in clinical depressive disorders cohorts during forest therapy is rare.

And the existing research lacks of well-defined “dosing,” which means the parameters of adequate exposure, such as duration, frequency, and intensity, are undefined, hindering clinical prescription and reproducibility (116). Moreover, there is also a lack of attention to moderators. Current research seldom investigates for whom forest therapy works best. Potential moderators like genetic profile, baseline inflammation, severity of depressive disorders, socioeconomic background, or prior relationship with nature remain largely unexamined.

Last but not least, this narrative synthesis also has limitations. In a narrative review, selecting and weighing evidence involves scholarly interpretation, unlike in a protocol-driven systematic review. While we employed systematic search elements, this approach is susceptible to selection bias. The available literature inevitably shapes the synthesis. The current evidence base is fragmented and uneven, which precludes definitive conclusions and necessitates the cautious, hypothesis-generating tone of this review. We hope to make it a more systematic process, but the literature in this field is too interdisciplinary to realize the goal. Therefore, we expect this review to improve further as more scientific, specific, and concrete evidence emerges.

### 5.3 Key priorities for future research

To move the field forward, research must evolve from proof-of-concept studies to rigorous, mechanism-informed, and practically relevant science. We propose four interconnected priorities. Firstly, establishing efficacy and isolating active ingredients through rigorous trials is essential. Researchers are advised to conduct large-scale, multicenter RCTs with active comparators, factorial designs to disentangle components, blinded outcome assessors, and long-term follow-up (6–12 months). And implementing dose–response studies to define minimum adequate exposure and optimal scheduling is also crucial for informing standardized prescription guidelines. Secondly, future research should focus on validating the multi-system mechanism model by employing experimental medicine approaches, in which participants undergo controlled forest exposures and undergo intensive longitudinal sampling of a multi-modal biomarker panel, including cortisol, HRV, inflammatory markers, BDNF, and potentially neuroimaging. Such studies can test the central hypothesis of synchronous multi-system change and identify which biomarker shifts best predict clinical improvement. Thirdly, we suggested that future studies integrate moderator analyses into clinical trials to identify subgroups of responders, for instance, based on inflammatory status, genetic markers, or specific depressive symptom profiles. And center environmental justice in research design is also needed to be added. And Study must guide policy to ensure benefits are equitably distributed, not a privilege of the affluent or geographically fortunate. Lastly, Future research should develop and evaluate feasible, scalable delivery models for different settings. We hope to strengthen interdisciplinary collaboration between public health, psychiatry, forestry, and landscape architecture to define the key biophysical characteristics of a therapeutic forest, enabling evidence-based design.

## 6 Conclusion

**Figure 2:**
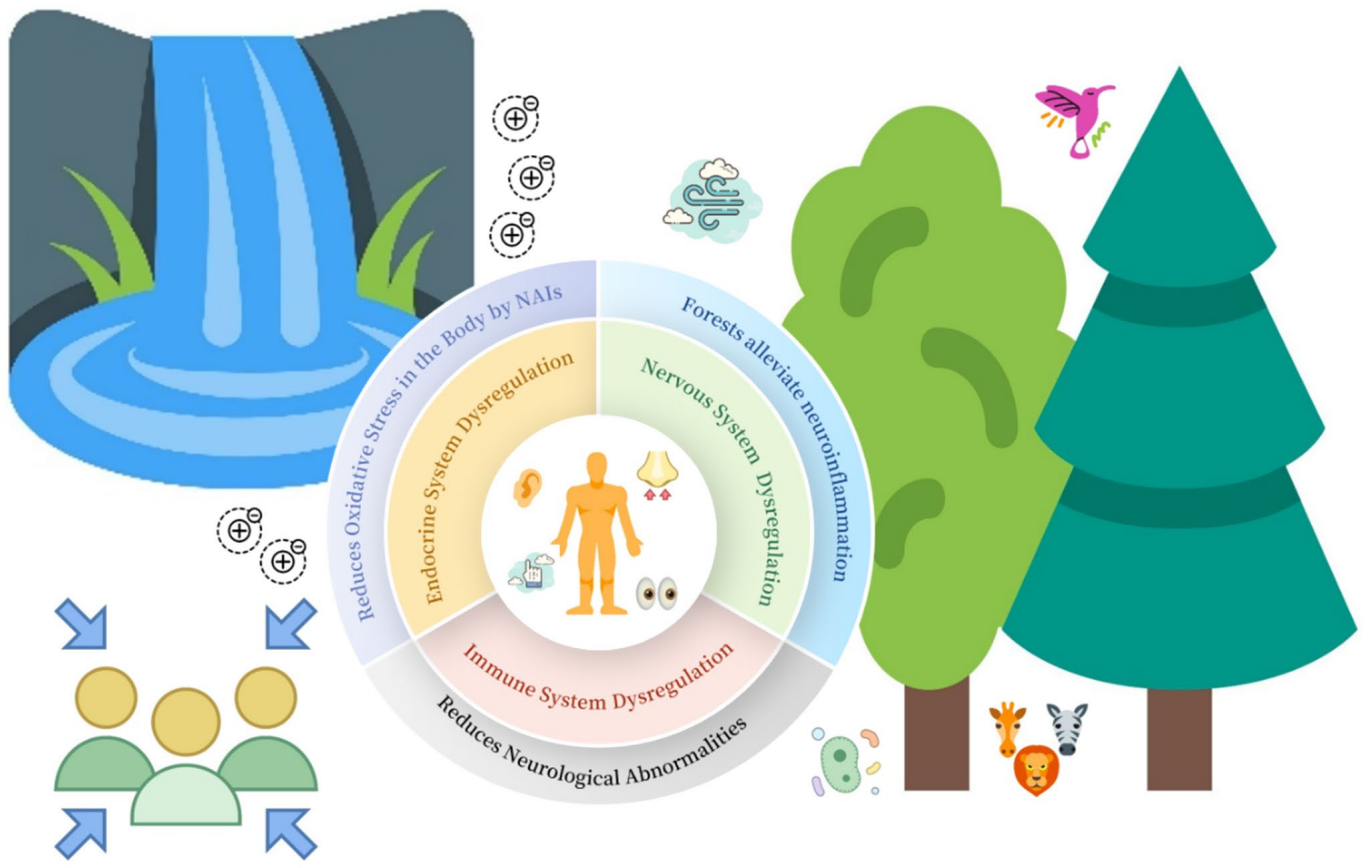
Integrated mechanism model of forest therapy as an adjunctive treatment for depressive disorders (self-drawn by the author).

This study synthesizes the mechanisms and pathways by which the forest environment influences human mental health, specifically targeting the pathological mechanisms of depressive disorder (Figure 2). Current epidemiological and environmental toxicology studies have yet to demonstrate the significant efficacy of forest therapy for depressive disorders conclusively. Furthermore, numerous studies have confirmed the critical role of neuroinflammation, oxidative stress, impaired neurogenesis, and apoptosis in the pathogenesis of depressive disorders, and a growing body of research has established that forest environments can modulate these physiological processes.

Forest therapy exemplifies a holistic, system-level approach to a complex, systemic illness. The current evidence, while preliminary, converges to support a multi-pathway, multi-target model of action that aligns well with the networked pathophysiology of depressive disorders. The challenge ahead is to replace enthusiasm with empirical precision. By embracing rigorous clinical science, advanced mechanistic studies, and a steadfast commitment to personalization and equity, forest therapy can evolve. The goal is not to replace conventional treatments but to establish its role as a credible, accessible, and effective component within integrative mental healthcare strategies, offering a unique path to healing that reconnects individuals with their own physiology, their social world, and the natural environment.

## References

[1] Chen X Li F Zuo H Zhu F. Trends in prevalent cases and disability-adjusted life-years of depressive disorders worldwide: findings from the global burden of disease study from 1990 to 2021. Depress Anxiety. (2025) 2025:5553491. doi: 10.1155/da/5553491, 40313474 PMC12045679

[2] Wild CP. The exposome: from concept to utility. Int J Epidemiol. (2012) 41:24–32. doi: 10.1093/ije/dyr236, 22296988

[3] Zhang Z Zhao Q Xu J Yang K Wu Y Lei M . Linking environmental exposures to depression: insights from epidemiology, biology, and methodology. Brain Conflux. (2025) 1:e244. doi: 10.71321/ymyc0370

[4] Van Den Bosch M Meyer-Lindenberg A. Environmental exposures and depression: biological mechanisms and epidemiological evidence. Annu Rev Public Health. (2019) 40:239–59. doi: 10.1146/annurev-publhealth-040218-044106, 30633709

[5] Nabeshima T Kim H-C. Involvement of genetic an d environmental factors in the onset of depression. Exp Neurobiol. (2013) 22:235–43. doi: 10.5607/en.2013.22.4.235, 24465138 PMC3897684

[6] Annavarapu RN Kathi S. Cognitive disorders in children associated with urban vehicular emissions. Environ Pollut. (2016) 208:74–8. doi: 10.1016/j.envpol.2015.09.036, 26476694

[7] Genc S Zadeoglulari Z Fuss SH Genc K. The adverse effects of air pollution on the nervous system. J Toxicol. (2012) 2012:782462. doi: 10.1155/2012/782462, 22523490 PMC3317189

[8] Yang J McBride J Zhou J Sun Z. The urban forest in Beijing and its role in air pollution reduction. Urban For Urban Green. (2005) 3:65–78. doi: 10.1016/j.ufug.2004.09.001

[9] Nowak DJ Hirabayashi S Bodine A Greenfield E. Tree and forest effects on air quality and human health in the United States. Environ Pollut. (2014) 193:119–29. doi: 10.1016/j.envpol.2014.05.028, 25016465

[10] Annerstedt M Währborg P. Nature-assisted therapy: systematic review of controlled and observational studies. Scand J Public Health. (2011) 39:371–88. doi: 10.1177/1403494810396400, 21273226

[11] Ulrich RS. View through a window may influence recovery from surgery. Science. (1984) 224:420–1. 6143402 10.1126/science.6143402

[12] Li Q Morimoto K Kobayashi M Inagaki H Katsumata M Hirata Y . Visiting a Forest, but not a City, increases human natural killer activity and expression of anti-Cancer proteins. Int J Immunopathol Pharmacol. (2008) 21:117–27. doi: 10.1177/039463200802100113, 18336737

[13] Li Q Morimoto K Nakadai A Inagaki H Katsumata M Shimizu T . Forest bathing enhances human natural killer activity and expression of anti-cancer proteins. Int J Immunopathol Pharmacol. (2007) 20:3–8. doi: 10.1177/03946320070200S202 17903349

[14] Li Q Nakadai A Matsushima H Miyazaki Y Krensky AM Kawada T . Phytoncides (wood essential oils) induce human natural killer cell activity. Immunopharmacol Immunotoxicol. (2006) 28:319–33. doi: 10.1080/08923970600809439, 16873099

[15] Cho KS Lim Y Lee K Lee J Lee JH Lee I-S. Terpenes from forests and human health. ToxicolRes. (2017) 33:97–106. doi: 10.5487/TR.2017.33.2.097, 28443180 PMC5402865

[16] Mitchell R. Is physical activity in natural environments better for mental health than physical activity in other environments? Soc Sci Med. (2013) 91:130–4. doi: 10.1016/j.socscimed.2012.04.012, 22705180

[17] Dadvand P Bartoll X Basagaña X Dalmau-Bueno A Martinez D Ambros A . Green spaces and general health: roles of mental health status, social support, and physical activity. Environ Int. (2016) 91:161–7. doi: 10.1016/j.envint.2016.02.029 26949869

[18] De Vries S Van Dillen SM Groenewegen PP Spreeuwenberg P. Streetscape greenery and health: stress, social cohesion and physical activity as mediators. Soc Sci Med. (2013) 94:26–33. doi: 10.1016/j.socscimed.2013.06.030, 23931942

[19] O’Brien L Morris J. Well-being for all? The social distribution of benefits gained from woodlands and forests in Britain. Local Environ. (2014) 19:356–83. doi: 10.1080/13549839.2013.790354

[20] Groenewegen PP Zock J-P Spreeuwenberg P Helbich M Hoek G Ruijsbroek A . Neighbourhood social and physical environment and general practitioner assessed morbidity. Health Place. (2018) 49:68–84. doi: 10.1016/j.healthplace.2017.11.006, 29227885

[21] Bratman GN Daily GC Levy BJ Gross JJ. The benefits of nature experience: improved affect and cognition. Landsc Urban Plan. (2015) 138:41–50. doi: 10.1016/j.landurbplan.2015.02.005

[22] Martens D. Bauer N. (2013). Natural Environments: a Resource for Public Health and Well-being? A Literature Review. In E. Noehammer (Ed.), Psychology of well-being: Theory, perspectives and practice (pp. 173–217). Nova Science Publishers.

[23] Meyer K Botsch K. Do forest and health professionals presume that forests offer health benefits, and is cross-sectional cooperation conceivable? Urban For Urban Green. (2017) 27:127–37. doi: 10.1016/j.ufug.2017.07.002

[24] O’Brien L Morris J Stewart A. Engaging with peri-urban woodlands in England: the contribution to people’s health and well-being and implications for future management. Int J Environ Res Public Health. (2014) 11:6171–92. doi: 10.3390/ijerph110606171, 24927035 PMC4078573

[25] Tyrväinen L Ojala A Korpela K Lanki T Tsunetsugu Y Kagawa T. The influence of urban green environments on stress relief measures: a field experiment. J Environ Psychol. (2014) 38:1–9. doi: 10.1016/j.jenvp.2013.12.005

[26] Lee J Tsunetsugu Y Takayama N Park B-J Li Q Song C . Influence of forest therapy on cardiovascular relaxation in young adults. Evid Based Complement Alternat Med. (2014) 2014:834360. doi: 10.1155/2014/834360, 24660018 PMC3934621

[27] Park BJ Tsunetsugu Y Kasetani T Kagawa T Miyazaki Y. The physiological effects of Shinrin-yoku (taking in the forest atmosphere or forest bathing): evidence from field experiments in 24 forests across Japan. Environ Health Prev Med. (2010) 15:18–26. doi: 10.1007/s12199-009-0086-9, 19568835 PMC2793346

[28] Berman MG Jonides J Kaplan S. The cognitive benefits of interacting with nature. Psychol Sci. (2008) 19:1207–12. doi: 10.1111/j.1467-9280.2008.02225.x, 19121124

[29] Hartig T Evans GW Jamner LD Davis DS Gärling T. Tracking restoration in natural and urban field settings. J Environ Psychol. (2003) 23:109–23. doi: 10.1016/S0272-4944(02)00109-3

[30] Laumann K Gärling T Stormark KM. Selective attention and heart rate responses to natural and urban environments. J Environ Psychol. (2003) 23:125–34. doi: 10.1016/S0272-4944(02)00110-X

[31] Keniger LE Gaston KJ Irvine KN Fuller RA. What are the benefits of interacting with nature? Int J Environ Res Public Health. (2013) 10:913–35. doi: 10.3390/ijerph10030913, 23466828 PMC3709294

[32] Jiang B Chang C-Y Sullivan WC. A dose of nature: tree cover, stress reduction, and gender differences. Landsc Urban Plan. (2014) 132:26–36. doi: 10.1016/j.landurbplan.2014.08.005

[33] Jiang B Li J Gong P Webster C Schumann G Liu X . A generalized relationship between dose of greenness and mental health response. Nature Cities. (2025) 2:739–48. doi: 10.1038/s44284-025-00285-z

[34] Zhou P Cheng Y Rosenberg MW. The concept of therapeutic landscape and its research progress in health geography. Prog Geogr. (2023) 42:602–16. doi: 10.18306/dlkxjz.2023.03.015

[35] Hamon M Blier P. Monoamine neurocircuitry in depression and strategies for new treatments. Prog Neuro-Psychopharmacol Biol Psychiatry. (2013) 45:54–63. doi: 10.1016/j.pnpbp.2013.04.009, 23602950

[36] Swaab DF Bao A-M Lucassen PJ. The stress system in the human brain in depression and neurodegeneration. Ageing Res Rev. (2005) 4:141–94. doi: 10.1016/j.arr.2005.03.003, 15996533

[37] Bollen J Trick L Llewellyn D Dickens C. The effects of acute inflammation on cognitive functioning and emotional processing in humans: a systematic review of experimental studies. J Psychosom Res. (2017) 94:47–55. doi: 10.1016/j.jpsychores.2017.01.002, 28183402

[38] Kraus C Castrén E Kasper S Lanzenberger R. Serotonin and neuroplasticity–links between molecular, functional and structural pathophysiology in depression. Neurosci Biobehav Rev. (2017) 77:317–26. doi: 10.1016/j.neubiorev.2017.03.007, 28342763

[39] Li M D’arcy C Meng X. Maltreatment in childhood substantially increases the risk of adult depression and anxiety in prospective cohort studies: systematic review, meta-analysis, and proportional attributable fractions. Psychol Med. (2016) 46:717–30. doi: 10.1017/S0033291715002743, 26708271

[40] Hyvönen K Salonen K Paakkolanvaara J-V Väkeväinen P Korpela K. Effects of nature-based intervention in the treatment of depression: a multi-center, randomized controlled trial. J Environ Psychol. (2023) 85:101950. doi: 10.1016/j.jenvp.2022.101950

[41] Owens M Bunce HL. The potential for outdoor nature-based interventions in the treatment and prevention of depression. Front Psychol. (2022) 13:740210. doi: 10.3389/fpsyg.2022.740210, 35401311 PMC8984301

[42] Rajoo KS Karam DS Abdullah MZ. The physiological and psychosocial effects of forest therapy: a systematic review. Urban For Urban Green. (2020) 54:126744. doi: 10.1016/j.ufug.2020.126744

[43] Kazlauckas V Pagnussat N Mioranzza S Kalinine E Nunes F Pettenuzzo L . Enriched environment effects on behavior, memory and BDNF in low and high exploratory mice. Physiol Behav. (2011) 102:475–80. doi: 10.1016/j.physbeh.2010.12.025, 21236277

[44] Khalil MH. The BDNF-interactive model for sustainable hippocampal neurogenesis in humans: synergistic effects of environmentally-mediated physical activity, cognitive stimulation, and mindfulness. Int J Mol Sci. (2024) 25:12924. doi: 10.3390/ijms252312924, 39684635 PMC11641763

[45] Li Q. New concept of forest medicine. Forests. (2023) 14:1024. doi: 10.3390/f14051024

[46] Vargas-Uricoechea H Castellanos-Pinedo A Urrego-Noguera K Vargas-Sierra HD Pinzón-Fernández MV Barceló-Martínez E . Mindfulness-based interventions and the hypothalamic–pituitary–adrenal axis: a systematic review. Neurol Int. (2024) 16:1552–84. doi: 10.3390/neurolint16060115, 39585074 PMC11587421

[47] Figueiredo Godoy AC Frota FF Araújo LP Valenti VE Pereira E d SBM Detregiachi CRP . Neuroinflammation and natural antidepressants: balancing fire with flora. Biomedicine. (2025) 13:1129. doi: 10.3390/biomedicines13051129, 40426956 PMC12108937

[48] Morris G Berk M Carvalho A Caso JR Sanz Y Walder K . The role of the microbial metabolites including tryptophan catabolites and short chain fatty acids in the pathophysiology of immune-inflammatory and neuroimmune disease. Mol Neurobiol. (2017) 54:4432–51. doi: 10.1007/s12035-016-0004-2, 27349436

[49] Choi H Jeon Y-H Han J-W Moon J Kim S-Y Woo J-M. The effects of a forest therapy on work-related stress for employees in the manufacturing industry: randomized control study. Global Advan Health Med. (2022) 11:2164957X221100468. doi: 10.1177/2164957X221100468

[50] Díaz-Martínez F Sánchez-Sauco MF Orenes-Piñero E Martínez-Romera I Ortega-García JA. Effects of forest therapy on salivary biomarkers (cortisol, amylase and IGA) in pediatric cáncer survivors: an experimental study. Anales de Pediatría (English Edition). (2023) 99:356–7. doi: 10.1016/j.anpede.2023.10.007, 39109640

[51] Hunter MR Gillespie BW Chen SY-P. Urban nature experiences reduce stress in the context of daily life based on salivary biomarkers. Front Psychol. (2019) 10:413490. doi: 10.3389/fpsyg.2019.00722 PMC6458297 31019479

[52] Jha MK Leboyer M Pariante CM Miller AH. Should inflammation be a specifier for major depression in the DSM-6? JAMA Psychiatry. (2025) 82:549–50. doi: 10.1001/jamapsychiatry.2025.0206, 40172869

[53] de la Pérez Mora M Borroto-Escuela DO Crespo-Ramírez M Rejón-Orantes J d C Palacios-Lagunas DA Martínez-Mata MK . Dysfunctional heteroreceptor complexes as novel targets for the treatment of major depressive and anxiety disorders. Cells. (2022) 11:1826. doi: 10.3390/cells11111826, 35681521 PMC9180493

[54] Fries GR Saldana VA Finnstein J Rein T. Molecular pathways of major depressive disorder converge on the synapse. Mol Psychiatry. (2023) 28:284–97. doi: 10.1038/s41380-022-01806-1, 36203007 PMC9540059

[55] Correia AS Vale N. Tryptophan metabolism in depression: a narrative review with a focus on serotonin and kynurenine pathways. Int J Mol Sci. (2022) 23:8493. doi: 10.3390/ijms23158493, 35955633 PMC9369076

[56] Lv J Wang W Krafft T Li Y Zhang F Yuan F. Effects of several environmental factors on longevity and health of the human population of Zhongxiang, Hubei, China. Biol Trace Elem Res. (2011) 143:702–16. doi: 10.1007/s12011-010-8914-8, 21153714

[57] Wu CC Lee GW Yang S Yu K-P Lou CL. Influence of air humidity and the distance from the source on negative air ion concentration in indoor air. Sci Total Environ. (2006) 370:245–53. doi: 10.1016/j.scitotenv.2006.07.020, 16916532

[58] Sudimac S. Exposure to Natural Versus Urban Environments: Short-Term Effects on Stress, Stress-Related Brain Function, and Hippocampal Structure. Germany: Freie Universitaet Berlin (2024).

[59] Lee S-H Sohn J-H Sung JH Han S-W Lee M Kim Y . The impact of forest therapy on functional recovery after acute ischemic stroke. Urban For Urban Green. (2024) 101:128537. doi: 10.1016/j.ufug.2024.128537

[60] Li Q. Effects of forest environment (Shinrin-yoku/Forest bathing) on health promotion and disease prevention—the establishment of “Forest medicine”—. Environ Health Prev Med. (2022) 27:43–3. doi: 10.1265/ehpm.22-00160, 36328581 PMC9665958

[61] Bayleyegn Derso T Mengistu BA Demessie Y Fenta MD Getnet K. Neural stem cells in adult neurogenesis and their therapeutic applications in neurodegenerative disorders: a concise review. Front Molecular Med. (2025) 5:1569717. doi: 10.3389/fmmed.2025.1569717, 40612293 PMC12222294

[62] Correia A Cardoso A Vale N. Bdnf unveiled: exploring its role in major depression disorder serotonergic imbalance and associated stress conditions. Pharmaceutics. (2023) 15:2081. doi: 10.3390/pharmaceutics15082081, 37631295 PMC10457827

[63] Dwivedi Y. Brain-derived neurotrophic factor: role in depression and suicide. Neuropsychiatr Dis Treat. (2009) 5:433–49. doi: 10.2147/NDT.S5700 19721723 PMC2732010

[64] Mazzocchi-Jones D Döbrössy M Dunnett SB. Environmental enrichment facilitates long-term potentiation in embryonic striatal grafts. Neurorehabil Neural Repair. (2011) 25:548–57. doi: 10.1177/1545968311402090, 21444652

[65] Silveira-Rodrigues JG Campos BT de Lima AT Ogando PH Gomes CB Gomes PF . Acute bouts of aerobic and resistance exercise similarly alter inhibitory control and response time while inversely modifying plasma BDNF concentrations in middle-aged and older adults with type 2 diabetes. Exp Brain Res. (2023) 241:1173–83. doi: 10.1007/s00221-023-06588-8, 36912948

[66] Shin M Shin C Lee M Lee J Choi A Choi J. Effects of exercise program with different forest slopes on BDNF and depression of women in 50s. J Korean Inst For Rec. (2015) 19:109–15. doi: 10.34272/forest.2015.19.1.011

[67] Alkadhi KA. Exercise as a positive modulator of brain function. Mol Neurobiol. (2018) 55:3112–30. doi: 10.1007/s12035-017-0516-4, 28466271

[68] Romero Garavito A Díaz Martínez V Juárez Cortés E Negrete Díaz JV Montilla Rodríguez LM. Impact of physical exercise on the regulation of brain-derived neurotrophic factor in people with neurodegenerative diseases. Front Neurol. (2025) 15:1505879. doi: 10.3389/fneur.2024.1505879, 39935805 PMC11810746

[69] El Hayek L Khalifeh M Zibara V Abi Assaad R Emmanuel N Karnib N . Lactate mediates the effects of exercise on learning and memory through SIRT1-dependent activation of hippocampal brain-derived neurotrophic factor (BDNF). J Neurosci. (2019) 39:2369–82. doi: 10.1523/JNEUROSCI.1661-18.2019, 30692222 PMC6435829

[70] Antonelli M Barbieri G Donelli D. Effects of forest bathing (shinrin-yoku) on levels of cortisol as a stress biomarker: a systematic review and meta-analysis. Int J Biometeorol. (2019) 63:1117–34. doi: 10.1007/s00484-019-01717-x, 31001682

[71] Sudimac S Sale V Kühn S. How nature nurtures: amygdala activity decreases as the result of a one-hour walk in nature. Mol Psychiatry. (2022) 27:4446–52. doi: 10.1038/s41380-022-01720-6, 36059042 PMC9734043

[72] Singh DK Kumar B Sinha S Fatima K. Phytochemicals for the improvement of cognitive function through cholinergic anti-inflammatory pathway. Current Indian Sci. (2024) 3:e2210299X309791. doi: 10.2174/012210299X309791240914125655

[73] Thangaleela S Sivamaruthi BS Kesika P Bharathi M Kunaviktikul W Klunklin A . Essential oils, phytoncides, aromachology, and aromatherapy—a review. Appl Sci. (2022) 12:4495. doi: 10.3390/app12094495

[74] Andersen L Corazon SS Stigsdotter UK. Nature exposure and its effects on immune system functioning: a systematic review. Int J Environ Res Public Health. (2021) 18:1416. doi: 10.3390/ijerph18041416, 33546397 PMC7913501

[75] Tsao T-M Tsai M-J Hwang J-S Cheng W-F Wu C-F Chou C-C . Health effects of a forest environment on natural killer cells in humans: An observational pilot study. Oncotarget. (2018) 9:16501–11. doi: 10.18632/oncotarget.24741, 29662662 PMC5893257

[76] Molska M Mruczyk K Cisek-Woźniak A Prokopowicz W Szydełko P Jakuszewska Z . The influence of intestinal microbiota on BDNF levels. Nutrients. (2024) 16:2891. doi: 10.3390/nu16172891, 39275207 PMC11397622

[77] Peng Z Hou T Yang K Zhang J Mao Y-H Hou X. Microecologics and exercise: targeting the microbiota–gut–brain Axis for central nervous system disease intervention. Nutrients. (2025) 17:1769. doi: 10.3390/nu17111769, 40507038 PMC12157277

[78] Knezevic E Nenic K Milanovic V Knezevic NN. The role of cortisol in chronic stress, neurodegenerative diseases, and psychological disorders. Cells. (2023) 12:2726. doi: 10.3390/cells12232726, 38067154 PMC10706127

[79] Zhou L Wang T Yu Y Li M Sun X Song W . The etiology of poststroke-depression: a hypothesis involving HPA axis. Biomed Pharmacother. (2022) 151:113146. doi: 10.1016/j.biopha.2022.113146, 35643064

[80] Meyer N Harvey AG Lockley SW Dijk D-J. Circadian rhythms and disorders of the timing of sleep. Lancet. (2022) 400:1061–78. doi: 10.1016/S0140-6736(22)00877-7, 36115370

[81] Qiu Q Yang L He M Gao W Mar H Li J . The effects of forest therapy on the blood pressure and salivary cortisol levels of urban residents: a meta-analysis. Int J Environ Res Public Health. (2022) 20:458. doi: 10.3390/ijerph20010458, 36612777 PMC9819785

[82] Shuda Q Bougoulias ME Kass R. Effect of nature exposure on perceived and physiologic stress: a systematic review. Complement Ther Med. (2020) 53:102514. doi: 10.1016/j.ctim.2020.102514, 33066853

[83] Zeng Y Chourpiliadis C Hammar N Seitz C Valdimarsdóttir UA Fang F . Inflammatory biomarkers and risk of psychiatric disorders. JAMA Psychiatry. (2024) 81:1118–29. doi: 10.1001/jamapsychiatry.2024.2185, 39167384 PMC11339698

[84] Yang Q Zhou J. Neuroinflammation in the central nervous system: symphony of glial cells. Glia. (2019) 67:1017–35. doi: 10.1002/glia.23571, 30548343

[85] Cai Y Liu J Wang B Sun M Yang H. Microglia in the neuroinflammatory pathogenesis of Alzheimer’s disease and related therapeutic targets. Front Immunol. (2022) 13:856376. doi: 10.3389/fimmu.2022.856376, 35558075 PMC9086828

[86] Ehsanifar M Montazeri Z Zavareh MS Rafati M Wang J. Cognitive impairment, depressive-like behaviors and hippocampal microglia activation following exposure to air pollution nanoparticles. Environ Sci Pollut Res. (2023) 30:23527–37. doi: 10.1007/s11356-022-23882-0, 36327074

[87] Ehsanifar M Tameh AA Farzadkia M Kalantari RR Zavareh MS Nikzaad H . Exposure to nanoscale diesel exhaust particles: Oxidative stress, neuroinflammation, anxiety and depression on adult male mice. Ecotoxicol Environ Saf. (2019) 168:338–47. doi: 10.1016/j.ecoenv.2018.10.090, 30391838

[88] Fonken LK Xu X Weil ZM Chen G Sun Q Rajagopalan S . Air pollution impairs cognition, provokes depressive-like behaviors and alters hippocampal cytokine expression and morphology. Mol Psychiatry. (2011) 16:987–95. doi: 10.1038/mp.2011.76, 21727897 PMC3270364

[89] Ji X Liu R Guo J Li Y Cheng W Pang Y . Olfactory bulb microglia activation mediated neuronal death in real-ambient particulate matter exposure mice with depression-like behaviors. Sci Total Environ. (2022) 821:153456. doi: 10.1016/j.scitotenv.2022.153456, 35093369

[90] Xiao S Wei T Petersen JD Zhou J Lu X. Biological effects of negative air ions on human health and integrated multiomics to identify biomarkers: a literature review. Environ Sci Pollut Res. (2023) 30:69824–36. doi: 10.1007/s11356-023-27133-8, 37170052 PMC10175061

[91] Antonelli M Donelli D Barbieri G Valussi M Maggini V Firenzuoli F. Forest volatile organic compounds and their effects on human health: a state-of-the-art review. Int J Environ Res Public Health. (2020) 17:6506. doi: 10.3390/ijerph17186506, 32906736 PMC7559006

[92] Kim T Song B Cho KS Lee I-S. Therapeutic potential of volatile terpenes and terpenoids from forests for inflammatory diseases. Int J Mol Sci. (2020) 21:2187. doi: 10.3390/ijms21062187, 32235725 PMC7139849

[93] Kasapis C Thompson PD. The effects of physical activity on serum C-reactive protein and inflammatory markers: a systematic review. J Am Coll Cardiol. (2005) 45:1563–9. doi: 10.1016/j.jacc.2004.12.077, 15893167

[94] Nimmo M Leggate M Viana J King J. The effect of physical activity on mediators of inflammation. Diabetes Obes Metab. (2013) 15:51–60. doi: 10.1111/dom.12156 24003921

[95] Rubio-Valles M Ramos-Jimenez A. Effects of aerobic exercise on sleep quality, insomnia, and inflammatory markers: a systematic review and meta-analysis. Curr Issues Mol Biol. (2025) 47:572. doi: 10.3390/cimb47070572, 40729041 PMC12293783

[96] Donoso F Cryan JF Olavarría-Ramírez L Nolan YM Clarke G. Inflammation, lifestyle factors, and the microbiome-gut-brain Axis: relevance to depression and antidepressant action. Clin Pharmacol Therapeutics. (2023) 113:246–59. doi: 10.1002/cpt.2581, 35278334 PMC10084001

[97] Padoan A Musso G Contran N Basso D. Inflammation, autoinflammation and autoimmunity in inflammatory bowel diseases. Curr Issues Mol Biol. (2023) 45:5534–57. doi: 10.3390/cimb45070350, 37504266 PMC10378236

[98] Kıran TR Otlu O Karabulut AB. Oxidative stress and antioxidants in health and disease. J Lab Med. (2023) 47:1–11. doi: 10.1515/labmed-2022-0108

[99] Kosenko EA Kaminsky YG Stavrovskaya IG Sirota TV Kondrashova MN. The stimulatory effect of negative air ions and hydrogen peroxide on the activity of superoxide dismutase. FEBS Lett. (1997) 410:309–12. 9237652 10.1016/s0014-5793(97)00651-0

[100] Kondrashove M Grigorenko EV Tikhonov A Sirota TV Temnov AV Stavrovskaja IG . The primary physico-chemical mechanism for the beneficial biological/medical effects of negative air ions. IEEE Trans Plasma Sci. (2000) 28:230–7. doi: 10.1109/27.842910

[101] Xiao J-L Liu H-Y Sun C-C Tang C-F. Regulation of Keap1-Nrf2 signaling in health and diseases. Mol Biol Rep. (2024) 51:809. doi: 10.1007/s11033-024-09771-4, 39001962

[102] Yu C Xiao J-H. The Keap1-Nrf2 system: a mediator between oxidative stress and aging. Oxidative Med Cell Longev. (2021) 2021:6635460. doi: 10.1155/2021/6635460, 34012501 PMC8106771

[103] Chu C Zhang H Cui S Han B Zhou L Zhang N . Ambient PM2. 5 caused depressive-like responses through Nrf2/NLRP3 signaling pathway modulating inflammation. J Hazard Mater. (2019) 369:180–90. doi: 10.1016/j.jhazmat.2019.02.026, 30776601

[104] Siomek A. NF-κB signaling pathway and free radical impact. Acta Biochim Pol. (2012) 59:323–31. doi: 10.18388/abp.2012_2116, 22855720

[105] Mahapatra C Thakkar R Kumar R. Modulatory impact of oxidative stress on action potentials in pathophysiological states: a comprehensive review. Antioxidants. (2024) 13:1172. doi: 10.3390/antiox13101172, 39456426 PMC11504047

[106] Kim S Jang YS Park E-C. Associations between social isolation, withdrawal, and depressive symptoms in young adults: a cross-sectional study. BMC Psychiatry. (2025) 25:1–12. doi: 10.1186/s12888-025-06792-6 40181348 PMC11966788

[107] Balciunaite K. (2024) Experiences of Change in Connectedness Through Forest Bathing Among Adults With Disabilities and/or Physical Health Difficulties. Available online at: https://uhra.herts.ac.uk/id/eprint/15979/

[108] Sonntag-Öström E Stenlund T Nordin M Lundell Y Ahlgren C Fjellman-Wiklund A . “Nature’s effect on my mind”–patients’ qualitative experiences of a forest-based rehabilitation programme. Urban For Urban Green. (2015) 14:607–14. doi: 10.1016/j.ufug.2015.06.002

[109] Abookire SA Ayala SG Shadick NA. Supporting wellness, resilience, and community with forest therapy. Global Advan Integrative Med Health. (2024) 13:27536130241246503. doi: 10.1177/27536130241246503, 38601344 PMC11005489

[110] Chen Y Ye Y Ye Y. Synergistic drive between local knowledge and landscape design: construction and empirical evidence of landscape design in-situ evaluation system for forest health bases. Buildings. (2025) 15:1917. doi: 10.3390/buildings15111917

[111] Peluso É d TP Blay SL. Public stigma in relation to individuals with depression. J Affect Disord. (2009) 115:201–6. doi: 10.1016/j.jad.2008.08.013, 18842306

[112] Meng F Wu Y Ma D Yang B Diao H Dong D . Environmental justice at a crossroads: examining the impact of vegetation and building patterns on road-sourced PM2. 5 dispersion and population exposure. Ecolog Front. (2025) 45:667–77. doi: 10.1016/j.ecofro.2024.12.011

[113] Kuo M. How might contact with nature promote human health? Promising mechanisms and a possible central pathway. Front Psychol. (2015) 6:1093. doi: 10.3389/fpsyg.2015.01093, 26379564 PMC4548093

[114] Li Q. Effect of forest bathing trips on human immune function. Environ Health Prev Med. (2010) 15:9–17. doi: 10.1007/s12199-008-0068-3, 19568839 PMC2793341

[115] Lyu B Zeng C Xie S Li D Lin W Li N . Benefits of a three-day bamboo forest therapy session on the psychophysiology and immune system responses of male college students. Int J Environ Res Public Health. (2019) 16:4991. doi: 10.3390/ijerph16244991, 31817971 PMC6950568

[116] White MP Alcock I Grellier J Wheeler BW Hartig T Warber SL . Spending at least 120 minutes a week in nature is associated with good health and wellbeing. Sci Rep. (2019) 9:7730. doi: 10.1038/s41598-019-44097-3, 31197192 PMC6565732

[117] Khalil MH. Green environments for sustainable brains: parameters shaping adaptive neuroplasticity and lifespan Neurosustainability—a systematic review and future directions. Int J Environ Res Public Health. (2025) 22:690. doi: 10.3390/ijerph22050690, 40427807 PMC12111129

[118] Huang X Hussain B Chang J. Peripheral inflammation and blood–brain barrier disruption: effects and mechanisms. CNS Neurosci Ther. (2021) 27:36–47. doi: 10.1111/cns.13569, 33381913 PMC7804893

[119] Yin Y Ju T Zeng D Duan F Zhu Y Liu J . “Inflamed” depression: a review of the interactions between depression and inflammation and current anti-inflammatory strategies for depression. Pharmacol Res. (2024) 207:107322. doi: 10.1016/j.phrs.2024.107322, 39038630

